# Genetic interactions between *Protein Kinase D* and *Lobe* mutants during eye development of *Drosophila melanogaster*

**DOI:** 10.1186/s41065-019-0113-9

**Published:** 2019-12-19

**Authors:** Dieter Maier, Anja C. Nagel, Anette Preiss

**Affiliations:** Universität Hohenheim, Institut für Genetik (240A), Garbenstr. 30, 70599 Stuttgart, Germany

**Keywords:** *Drosophila melanogaster*, Protein kinase D, *PKD* null mutant, *Lobe*, Eye development

## Abstract

**Background:**

In *Drosophila,* the development of the fly eye involves the activity of several, interconnected pathways that first define the presumptive eye field within the eye anlagen, followed by establishment of the dorso-ventral boundary, and the regulation of growth and apoptosis. In *Lobe (L)* mutant flies, parts of the eye or even the complete eye are absent because the eye field has not been properly defined. Manifold genetic interactions indicate that *L* influences the activity of several signalling pathways, resulting in a conversion of eye tissue into epidermis, and in the induction of apoptosis. As information on the molecular nature of the *L* mutation is lacking, the underlying molecular mechanisms are still an enigma.

**Results:**

We have identified Protein Kinase D (PKD) as a strong modifier of the *L* mutant phenotype. PKD belongs to the PKC/CAMK class of Ser/Thr kinases that have been involved in diverse cellular processes including stress resistance and growth. Despite the many roles of PKD, *Drosophila PKD* null mutants are without apparent phenotype apart from sensitivity to oxidative stress. Here we report an involvement of *PKD* in eye development in the sensitized genetic background of *Lobe*. Absence of *PKD* strongly enhanced the dominant eye defects of heterozygous *L*^*2*^ flies, and decreased their viability. Moreover, eye-specific overexpression of an activated isoform of PKD considerably ameliorated the dominant *L*^*2*^ phenotype. This genetic interaction was not allele specific but similarly seen with three additional, weaker *L* alleles (*L*^*1*^*, L*^*5*^*, L*^*G*^), demonstrating its specificity.

**Conclusions:**

We propose that PKD-mediated phosphorylation is involved in underlying processes causing the *L* phenotype, i.e. in the regulation of growth, the epidermal transformation of eye tissue and apoptosis, respectively.

## Background

**Fig. 1:**
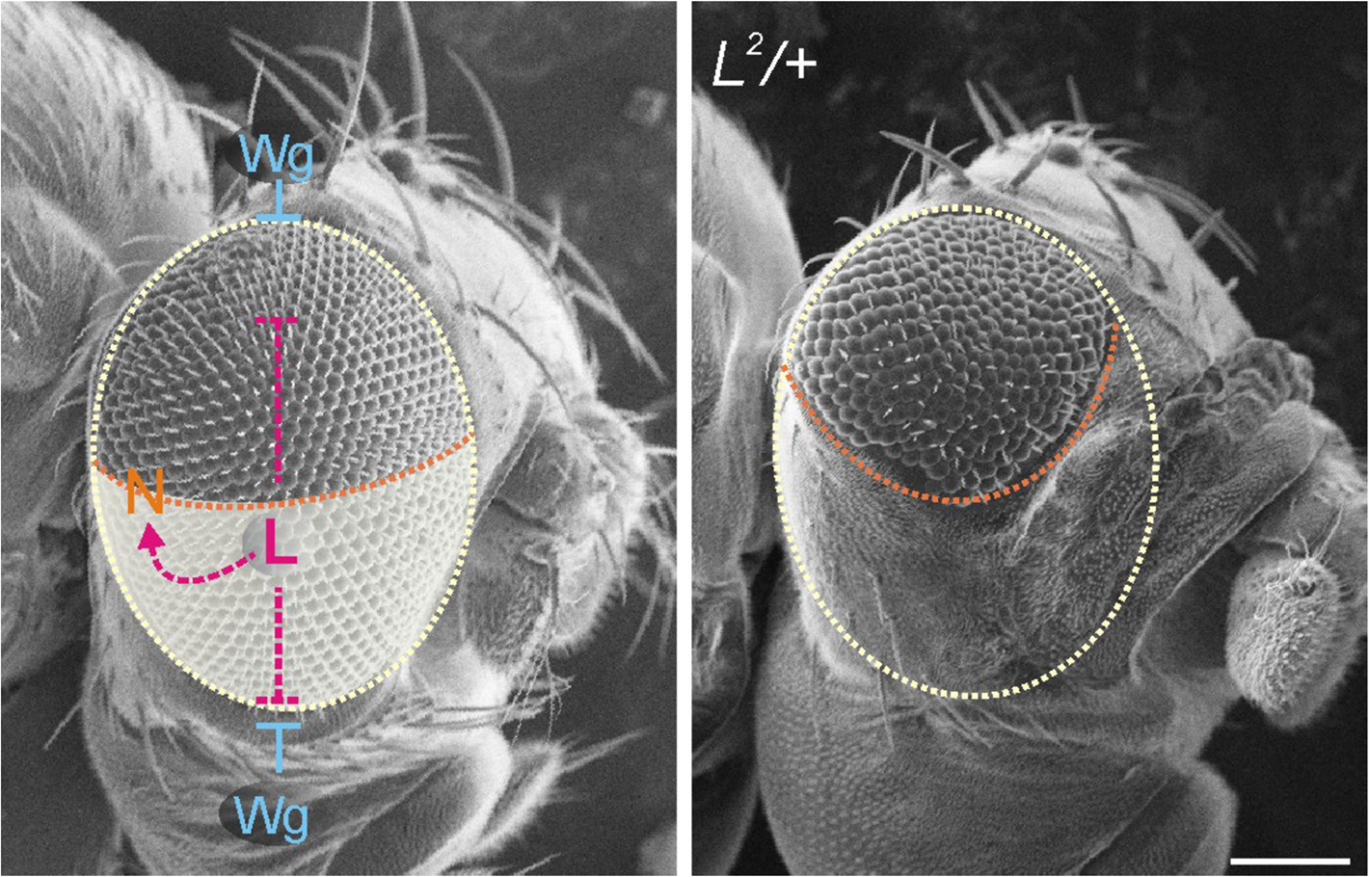
Development of the ventral eye is affected by the *L* mutation. During development, the *Drosophila* eye field is confined by the activity of Wg. Establishment of the dorso-ventral boundary requires the activation of N. Concluded from genetic interactions, *Lobe* acts as a positive regulator of N and a negative regulator of Wg activities. Accordingly, *L*^*2*^**/+** mutant flies display small eyes, where the ventral eye compartment is reduced at the expense of head epidermis. Arrows indicate positive, and bars negative interactions. Size bar, 100 μm

The *Drosophila* eye develops from the eye-antennal imaginal disc that eventually gives rise to the eye, the antenna and large parts of the head capsule (overview in: [1–3]). The master control genes for eye development in all seeing animals studied to date are encoded by the *Paired box 6 (Pax6)* gene family (overview in: [3–5]). The founding members in *D. melanogaster, eyeless (ey)* and *twin of eyeless (toy)*, are at the top of a network of retinal determination genes required for growth and the specification of the presumptive retinal field within the eye anlagen. One major task thereby is the suppression of non-ocular selector genes within the eye field, which otherwise direct the formation of head epidermis (overview in: [3]). Accordingly, retinal identity is lost in *ey* mutants accompanied by massive cell death, and *ey; toy* double mutants are even headless [6–10] (overview in: [3]). Several signalling pathways coordinate eye development, including the Wingless (Wg) and Notch (N) pathways. Whereas Wg is important for confining the presumptive eye field within the eye anlagen, N activity is required for establishing the dorso-ventral boundary as the major growth center (Fig. 1) (overview in: [2, 3]). Early on, the eye primordium is of ventral fate; formation of the dorsal compartment requires Wg signalling activity, which eventually triggers the activation of N along the midline (Fig. 1) [11–15].

In *Lobe (L)* mutant flies, eye development is disturbed: notably the ventral eye field is affected and in extreme cases, the complete eye is lacking (Fig. 1) [16]. Generally, the phenotypic character is very variable, and *L* mutant flies typically are strongly asymmetric. Whereas most *L* alleles overlap wild type in heterozygosis, and display reduced eyes only in homozygosis, the most extreme allele *L*^*2*^ is fully dominant and of poor viability in homozygosis [16]. Genetically, *L*^*2*^ is a neomorphic, dominant mutation since the mutant eye phenotype is reverted by deletions. Revertants are homozygous lethal at embryonic stage, but the corresponding gene has not been identified so far [17, 18]. *L* defects can be traced to the early development of the eye precursor: Fewer cells enter into eye disc formation, and the cephalic complex is significantly reduced in size already in first instar larva [16, 18–21]. *L* has been tightly linked to Wg and N signalling pathways in opposing ways [18, 22, 23]. With regard to N activity, *L* acts as a positive factor, being involved in the regulation of growth and survival of cells in the ventral compartment [18, 23]. In contrast, Wg and *L* act antagonistically. For example, the *L* mutant eye phenotype is enhanced by increased Wg activity, whereas it is ameliorated by *wg* mutants or by the overexpression of the Ser/Thr kinase Shaggy (Sgg), a known antagonist of Wg signalling [23]. In fact Wg is upregulated in ventral eye tissue mutant for *L*, resulting in a conversion of eye tissue into epidermis and induction of apoptosis (Fig. 1) [23–25].

Interestingly, the *L* mutant phenotype is exquisitely sensitive to genetic background [16, 21, 22]. For example, it is enhanced in a *Minute* background like the M (3)95A mutation, i.e. by the general downregulation of protein synthesis through mutation of ribosomal proteins like RpS3 [26, 27], suggesting a role for *L* in growth regulation. The proposed link between *L* and the negative TOR- regulator *Pras40*, however, has not been confirmed [28–30]. To date the molecular basis of the *L* mutation is still unknown. Hence, the molecular mechanisms underlying the *L* phenotype remain an enigma.

Here we show that Protein Kinase D (PKD) is a strong modifier of the *L* mutant phenotype. PKD is a member of the PKC/CAMK family of Ser/Thr kinases, and has been involved in diverse cellular processes including growth regulation, protection from oxidative stress, Golgi-mediated protein transport, as well as regulation of actin cytoskeletal dynamics in mammals (reviewed in: [31–34]), as well as in *Drosophila* [35–39]. Despite these many roles, *Drosophila PKD* null mutants are without apparent external phenotypes [40]. Moreover, mutant flies are not significantly different from control regarding fertility, longevity, growth and resistance to a variety of stressors including starvation apart from sensitivity to oxidative stress [40]. Apparently, PKD acts redundantly with other kinase members of the PKC/CAMK family, including PKCδ, Sqa and Drak [40]. In the course of the genetic combination of the *PKD* mutant with kinase candidates, we noted an unexpected, strong genetic interaction between the null allele *PKD*^*26*^ and *L*^*2*^: the dominant mutant eye phenotype was strongly enhanced, and viability of *L*^*2*^ heterozygotes markedly decreased. Specificity of the genetic interaction was confirmed by a rescue of the *L*^*2*^ eye phenotype through eye-specific overexpression of the activated isoform of PKD, PKD-SE. Finally, we show that this interaction is not allele specific but similarly seen with three additional, weaker *L* alleles (*L*^*1*^*, L*^*5*^*, L*^*G*^). We propose that PKD-mediated phosphorylation is involved in the molecular mechanisms underlying the aberrant eye development in *L* mutants.

## Results

### The small eye phenotype of *L*^*2*^ is controlled by protein kinase D activity

**Fig. 2:**
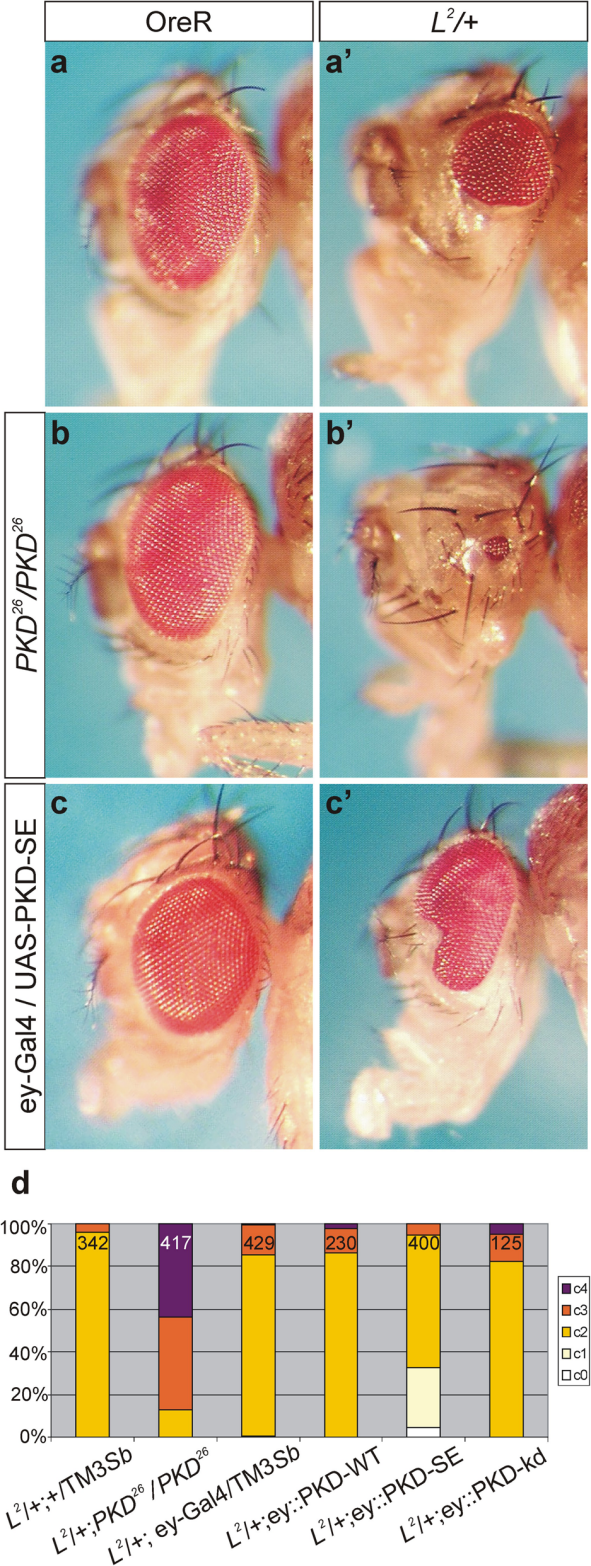
Genetic interaction of *PKD* with *L*^*2*^. Head of female flies; the left column shows a wild type OreR background (**a**), the right column a *L*^*2*^*/+* heterozygous background (a’). *PKD*^*26*^ homozygotes have wild type eyes (**b**). Yet, lack of *PKD* strongly enhances the dominant *L*^*2*^ small eye phenotype (b’). Overexpression of UAS-PKD-SE in the developing eye with ey-Gal4 is itself without apparent phenotype (**c**), but rescues the small eye phenotype of *L*^*2*^**/+** mutant flies (c’). (**d**) The dominant *L*^*2*^ eye phenotype is background-dependent and often varies between both eyes. For a quantitative analysis of the genetic interaction, the *L*^*2*^ eye phenotype was classified as follows: c0, wild type eyes; c1, one eye is smaller; c2, both eyes are smaller; c3, one eye is smaller and one is of pinhead size or absent; c4 both eyes are of pinhead size or absent. Genotypes are depicted below each column. The number of flies of the given genotype assessed in the experiment is indicated within each column. Note the strong enhancement of the *L*^*2*^ /+ phenotype by the absence of *PKD* and its rescue by overexpression of PKD-SE. Overexpression of PKD-WT had no impact, and that of PKD-kd caused little enhancement

In our conditions, the vast majority of *L*^*2*^ heterozygotes displayed an intermediate phenotype where both eyes are smaller (representative examples are shown in Fig. 1 and Fig. 2a, a’). In a *PKD* null mutant genetic background, i.e. combined with homozygous *PKD*^*26*^, the phenotype was strongly enhanced, since most flies displayed one or both eyes of pinhead size or complete absence (Fig. 2a’, b’). To account for the high phenotypic variability between the two eyes, and to allow a quantification of the genetic influence of *PKD* on *L*, we classified the phenotypes into five groups. *L*^*2*^ heterozygotes predominantly fell in class 2 (c2) (Fig. 2b’, d). Yet, in the absence of *PKD* nearly 90% of the flies were grouped into class 3 or 4 (c3, c4) respectively, i.e. no or little leftovers of one or both eyes (Fig. 2b’, d), in agreement with a strong requirement of PKD activity during *L*-dependent eye development.

It is well known that the *L* phenotype is exquisitely sensitive to environmental and genetic background [16, 21, 22]. As the *PKD*^*26*^ allele was generated by homologous recombination [40], the parental background cannot be easily reconstituted. Hence, other modifiers elsewhere on the third chromosome might be responsible for the phenotypic enhancement of *L*^*2*^ [21]. If, however, *PKD* were the culprit, overexpression of an activated form of the kinase would be expected to cause the opposite result, i.e. a rescue of the eye defects. Accordingly, the wild type PKD isoform should have little influence, as PKD activity requires its phosphorylation by upstream activating kinases [32], whereas a dominant negative isoform may enhance the *L*^*2*^ mutant eye phenotype. To confirm the specific role of *PKD*, we overexpressed the activated PKD-SE form specifically in the developing eye tissue, using the Gal4/UAS-system [36, 41]. To this end, *L*^*2*^ was combined with either ey-Gal4 [42], or with UAS-PKD-SE [36]. Whereas overexpression of UAS-PKD-SE in the eye anlagen does not alter the eye morphology (Fig. 2c), the offspring resulting from the subsequent cross indeed displayed a much milder phenotype: the eyes were generally larger (class1, kidney shaped, instead of class2, halved) (Fig. 2a’, c’), and frequently, only one of the two eyes was affected. The apparent impression was confirmed by a quantification of the results: a third of the flies was clearly rescued compared to their siblings (Fig. 2d). Similarly, UAS-PKD-WT and UAS-PKD-kd, encoding a wild type and presumptive ‘kinase dead’ PKD isoform, respectively [36], were overexpressed in the eye anlagen of *L*^*2*^ mutant larvae, and the resultant phenotypes were quantified (Fig. 2d). Compared to the control *L*^*2*^ /+; ey-Gal4/+ flies, overexpression of UAS-PKD-WT had little impact on eye development, whereas UAS-PKD-kd caused a slight enhancement, as predicted (Fig. 2d). These results clearly demonstrate the strong and specific influence of PKD activity on eye development in the *L*^*2*^ mutant background.

### Viability of *L*^*2*^ is impaired by the absence of *PKD*

**Fig. 3:**
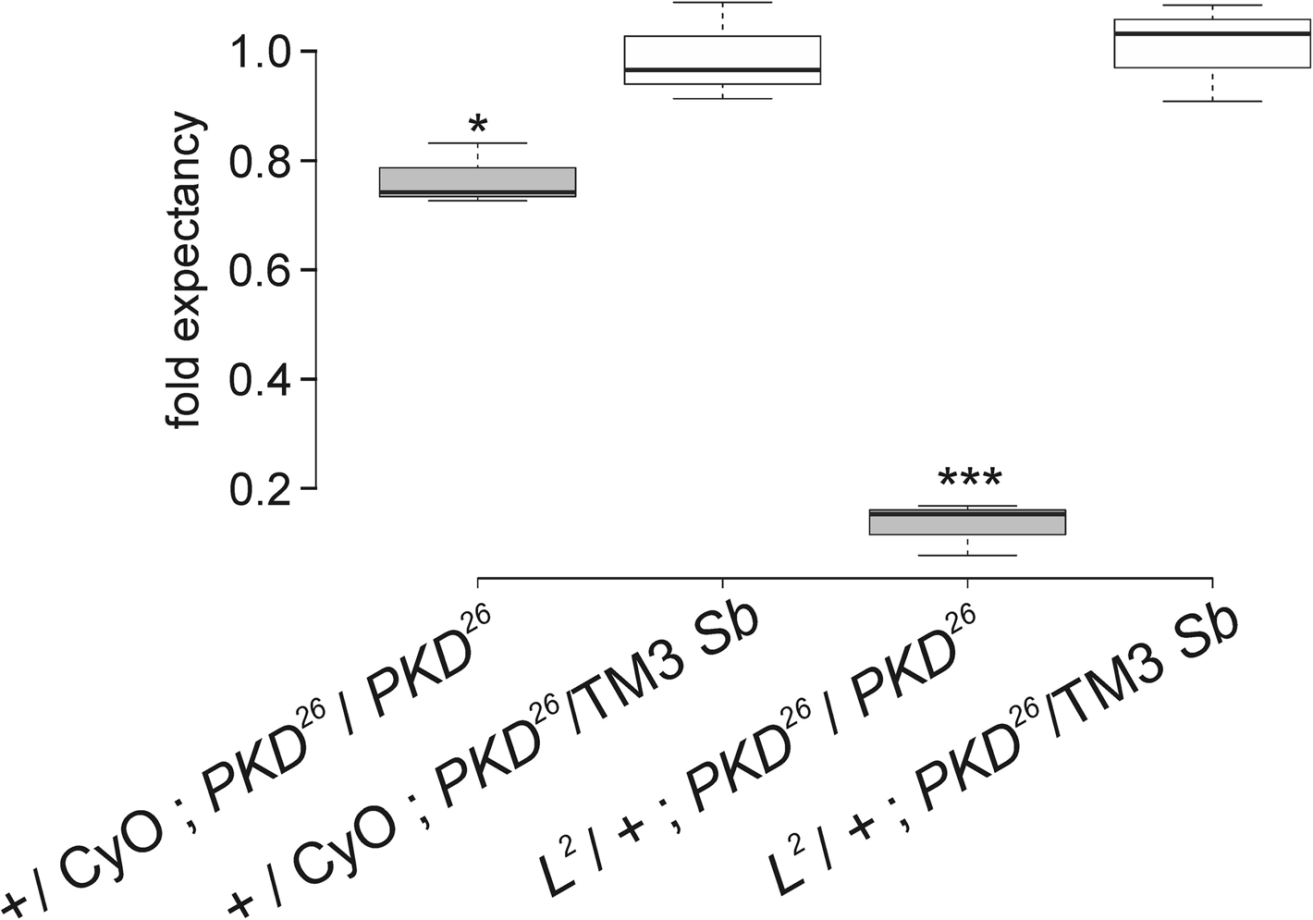
Viability of *L*^*2*^ heterozygotes is lowered in the absence of *PKD*. Viability of *PKD*^*26*^ homozygotes (labelled grey) in a *L*^*2*^ / + heterozygous background, compared to the doubly heterozygous siblings derived from a cross of *L*^*2*^ / *CyO*; *PKD*^*26*^/TM3 *Sb* males with virgins *PKD*^*26*^/ *PKD*^*26*^. Each genotype is expected with the same frequency; the *PKD*^*26*^ homozygotes however, reach only about 12% expectancy in the background of *L*^*2*^ (0.12). Center lines of BoxPlots display the median, box limits indicate the 25th and 75th percentiles as determined by R-software, whiskers extend 1.5 times the interquartile range. Three experiments were performed with 333 total number of flies. Statistical significance of probes was determined by ANOVA two-tailed test for multiple comparisons using Dunnett’s approach relative to the doubly heterozygous control, with raw *p*-values: *p* > 0.05 (not significant); * *p* < 0.05; ** *p* < 0.01; *** *p* < 0.001

In the course of the above experiments we noted that the number of *L*^*2*^ heterozygous flies lacking *PKD* (i.e. *L*^*2*^/+; *PKD*^*26*^/*PKD*^*26*^) was considerably lower compared to the siblings. The aberrancy was determined by quantifying the offspring from a cross of *L*^*2*^/*CyO*; *PKD*^*26*^/TM3 *Sb* males with virgins *PKD*^*26*^/*PKD*^*26*^. Four equal fractions with either homo- or heterozygous *PKD*^*26*^ genotype were expected. The homozygous *PKD*^*26*^ fraction carrying the *L*^*2*^ allele in one copy, however, reached only 12% of the expectation (Fig. 3). Apparently, viability of flies lacking PKD function is strongly impaired in the presence of the *L*^*2*^ allele.

### Genetic interactions between *PKD* and *Lobe* are not allele specific

**Fig. 4:**
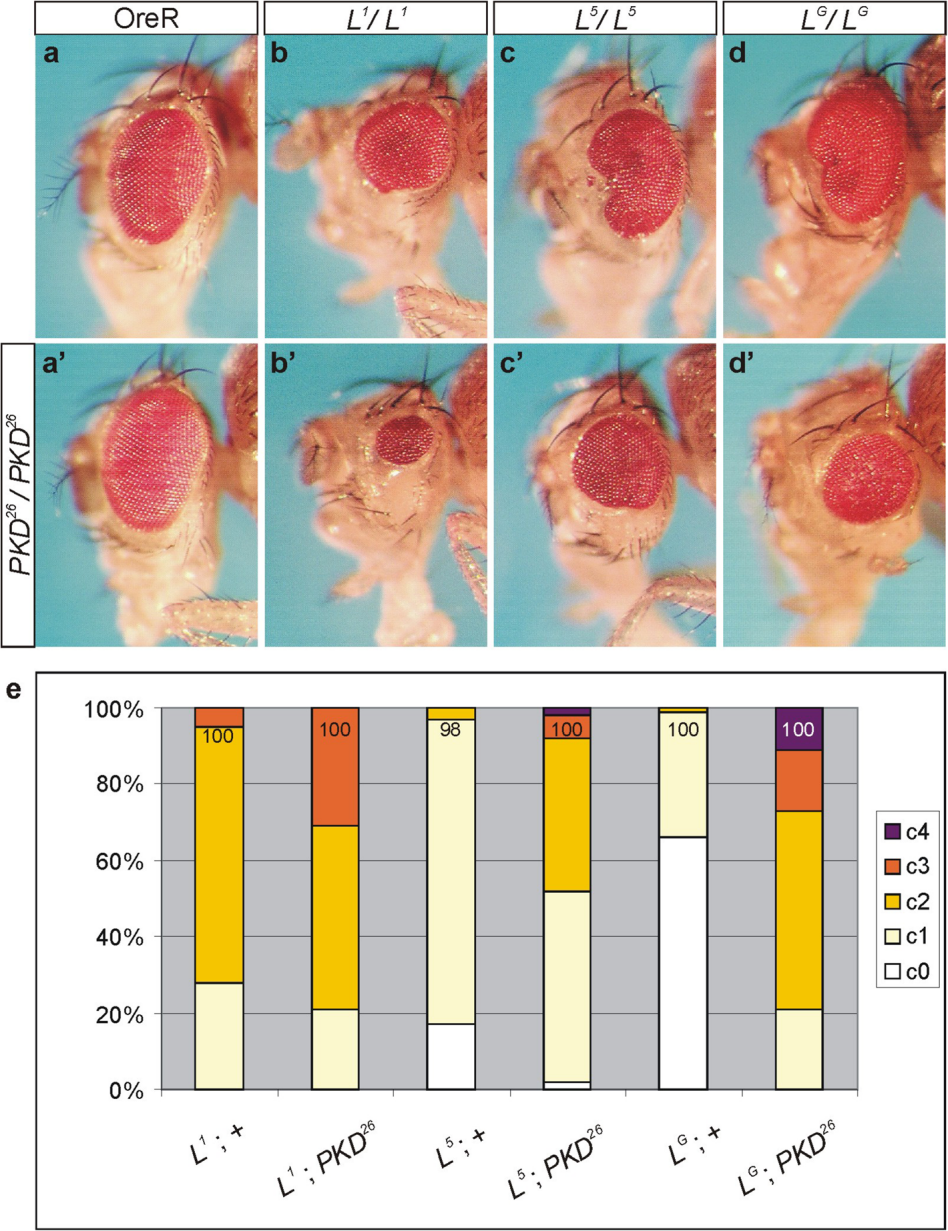
Phenotypic enhancement of recessive *L* alleles by the absence of *PKD*. Typical examples of female heads are shown. The upper row represents OreR as control (**a**) and three homozygous *Lobe* alleles*, L*^*1*^ (**b**)*, L*^*5*^ (**c**) and *L*^*G*^ (**d**). The lower row shows a typical example of a *PKD*^*26*^ homozygous mutant female (a’) in the above genetic background (b’-d’): note enhancement of the *L* small eye phenotype in the absence of PKD, independent of the *L* allele. (**e**) Quantification of eye phenotypes in the given genotype by using the following classification: c0, wild type eyes; c1, one eye is smaller; c2, both eyes are smaller; c3, one eye is smaller and one is of pinhead size or absent; c4 both eyes are of pinhead size or absent. Genotypes are depicted below each column; (+) indicates third chromosome normal for *PKD*. Crosses were performed at 25 °C. Fractions of phenotypes are depicted; numbers of assessed females are indicated in each column

*L*^*2*^ is the most extreme *Lobe* allele available with a fully penetrant dominant eye phenotype, and strongly reduced viability in the homozygotes. Other *L* alleles are generally weaker, and the heterozygous phenotype overlaps wild type [16]. In homozygosis, the alleles *L*^*1*^*, L*^*5*^ and *L*^*G*^ are viable and display variably strong eye defects (Fig. 4a-d) [16]. In our hands, *L*^*1*^ homozygotes displayed a fully penetrant phenotype, with the majority of flies developing strongly reduced eyes (Fig. 4b, e). The two other alleles were weaker: about 20% of the *L*^*5*^ and more than 60% of the *L*^*G*^ homozygotes overlapped wild type at 25 °C (Fig. 4c-e); eye defects were generally restricted to kidney shaped incisions (Fig. 4c, d). As the *L* phenotype is highly variable, it is difficult to generate a robust phenotypic series [16]. In the *PKD*^*26*^ homozygous background, however, the phenotypes were considerably enhanced; up to 30% of the flies now displayed only pinhead eye size, or lacked one or both eyes altogether (Fig. 4a’-d’, e). Interestingly, absence of *PKD* had the most obvious impact on the weakest allele *L*^*G*^, and the doubly homozygotes displayed as strong phenotypes as the *L*^*1*^;*PKD*^*26*^ combination (Fig. 4b’, d’, e).

**Fig. 5:**
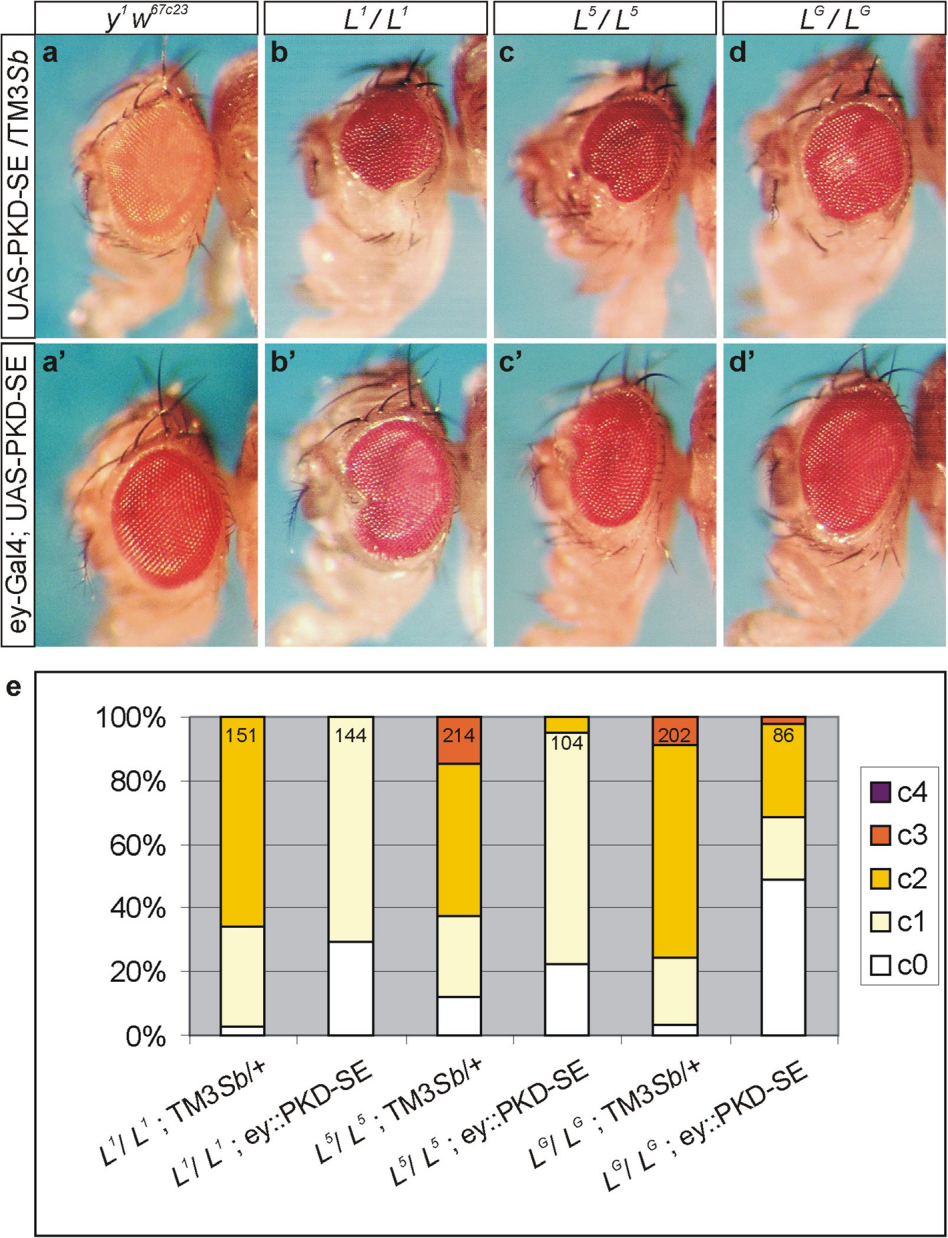
Overexpression of PKD-SE rescues the small eye phenotype of several *L* alleles. Typical examples of female heads are shown. (**a**-**d**) Upper row: Eye phenotype of the three homozygous *Lobe* alleles*, L*^*1*^ (**b**)*, L*^*5*^ (**c**)*,* and *L*^*G*^ (**d**) in the background of UAS-PKD-SE, which does not affect the eye of the control *y*^*1*^ *w*^*67c23*^ (**a**). (a’-d’) Lower row: Overexpression of UAS-PKD-SE with ey-Gal4 (ey::PKD-SE) does not disturb outer eye morphology (a’), however, results in a rescue of the small eye of any tested *L* allele (b’-d’). (**e**) Quantification of eye phenotypes in the given genotype, using the following classification: c0, wild type eyes; c1, one eye is smaller; c2, both eyes are smaller; c3, one eye is smaller and one is of pinhead size or absent; c4 both eyes are of pinhead size or absent. Genotypes are depicted below each column; (+) indicates third chromosome normal for *PKD*. Fractions of phenotypes are depicted; numbers of assessed females are indicated in each column. The animals were derived from a cross of L*/CyO; ey-Gal4/TM3*Sb* x L*/CyO; UAS-PKD-SE/TM3*Sb* at 18 °C

We again assessed the effect of the activated form of PKD on the extent of the *L* small eye phenotype in the background of the three different *L* alleles (Fig. 5). To this end, each *L* allele was combined with either UAS-PKD-SE or with ey-Gal4. The resultant flies were crossed, and their offspring analysed for eye defects (Fig. 5a-d’). As observed for *L*^*2*^, PKD-SE markedly rescued the recessive eye phenotype of either *L*^*1*^*, L*^*5*^ or *L*^*G*^ (Fig. 5e), confirming the specificity of the genetic interaction between the two genes. Again, *L*^*G*^ appeared most susceptible to the influence of PKD, since nearly half of the flies were phenotypically wild type (Fig. 5d’, e).

## Discussion

Our work identified striking genetic interactions between *PKD*^*26*^ and *Lobe* mutants, as the small eye phenotype of several *L* alleles was strongly enhanced in the absence of *PKD*, and ameliorated by activated PKD-SE overexpressed within the eye anlagen. Moreover, lethality of *L*^*2*^ heterozygotes was markedly increased in the *PKD*^*26*^ mutant background. Overall, our data reveal a requirement of PKD activity for eye development and fly survival, uncovered in the sensitized genetic background of *Lobe*. Despite the fact that PKD has been involved in rhodopsin Rh1 homeostasis in the adult retina, eye morphogenesis is unaffected in the mutants [36, 43]. Being a member of PKC/CAMK family of Ser/Thr kinases kinases, PKD most likely regulates both aspects of development, eye development and fly viability, by specific protein phosphorylation. Interestingly, the genetic link between *L* and several signalling pathways frequently involves Ser/Thr kinases or components that are phospho-targets thereof. Examples for respective kinases are *hemipterous*, a component of MAPK and JNK signalling, and *shaggy*, a negative regulator of Wg signalling activity, that modify the *L* phenotype when mutant [22, 23]. *L* interactors that are themselves phospho-targets are involved in MAPK-, JNK-, Wg-, Hh-, Dpp-, N-, and Jak/Stat-signalling pathways [22, 23], i.e. in the regulation of cell growth and apoptosis, and ultimately in eye development. Most likely PKD feeds into these pathways either directly or by shared phospho-targets.

A striking feature of the *L* mutant phenotype is the asymmetry, i.e. one eye may be lacking whereas the other may be nearly normal [16, 19, 21]. Similar defects are found in flies mutant for *eyeless (ey)* [6, 16, 44–47], the master regulator of eye development encoding the *Drosophila Pax6* homologue (overview in: [4, 5, 48]). The major role of *ey* is to control eye specification, and the survival and proliferation of eye progenitor cells [10, 45–47]. Similar to *L*, the *ey* mutant phenotype results from massive cell death within the eye anlagen of young larvae [7, 8, 45]. In fact, inhibition of apoptosis largely rescued lethality of *ey*^*D*^ mutants, resulting in completely eyeless adults [9]. Loss of the ventral eye field in *L* is likewise rescued by increased levels of cell death inhibitors or a downregulation of pro-apoptotic genes, indicating that apoptosis is a predominant trigger for the *L* eye defects [23]. In vertebrates, the requirement for PKD in the regulation of apoptosis is well established [32, 49]. Perhaps *PKD*, in concert with *L*, normally acts as a survival signal during early eye development. Normal flies are strikingly symmetrical with little size-differences between left and right, despite the fact that the two body parts and their appendages grow independently as imaginal discs to be fused only during pupal development [1]. This is also true for the two wild type eyes that show only small differences in the number of their ommatidia [50]. The *Drosophila* insulin like peptide Dilp8 mediates the homeostatic regulation through the coordination of growth of imaginal discs. It triggers a neurosecretory circuit by activating its receptor Lgr3 (Leucine-rich repeat containing G protein-coupled receptor 3) in a pair of neurons in the brain that act on downstream neuroendocrine cells. Eventually, growth of imaginal tissues is synchronized to maintain size proportions, thereby ensuring bilateral symmetry [51–55]. The striking asymmetry characterizing both *L* and *ey* mutants indicates a failure of coordinate growth regulation. Both, *L* and *ey* contribute to cell proliferation in the eye anlagen [10, 18, 23]. Moreover, *ey* controls the differentiation and function of insulin-producing cells within the larval brain, and thereby systemic growth of the whole animal [56]. Albeit a link to the Dilp8-mediated neurosecretory circuit has not yet been established, it clearly must integrate organ growth and systemic growth coordination [55, 57]. We speculate that it may also involve the activity of PKD.

Our recent work shows that *PKD* is dispensable for normal fly development, however, presumably acts redundantly with other members of the PKC/CAMK family. Three kinases, Pkcδ, Drak, and Sqa were uncovered as candidates for functionally redundant kinases [40]. In mammals, PKD and Pkcδ act in combatting oxidative stress (overview in: [34, 58]), conforming to a similar role for PKD in the fly [40]. Drak has an important role in epithelial tissue morphogenesis in *Drosophila*, in agreement with the involvement of PKD in the regulation of cytoskeletal dynamics [59, 60]. Perhaps, Drak and PKD act in the regulation of eye to epithelial transformation as well, which is defective in *L* mutants [25]. Finally, Sqa has a role in starvation-induced autophagy and the regulation of TOR signalling activity, linking PKD to growth control [61, 62]. As long as the nature *L* mutation remains unknown, however, we unfortunately can only speculate as to the molecular basis of the *PKD-L* interaction.

## Conclusion

The Ser/Thr kinase PKD is required for the regulation of growth and cell survival during eye development, which is uncovered by the sensitized background of the *L* mutation. Both, the fact that the *PKD*-*L* interaction is not allele specific and that it is bidirectional, i.e. enhancement of *L* in the absence of *PKD* and rescue of *L* by overactivity of *PKD*, supports the specificity of the genetic interaction. Most likely, PKD-mediated protein phosphorylation is involved in underlying molecular processes causing the *L* phenotype, i.e. in the regulation of growth, the epidermal transformation of eye tissue and apoptosis, respectively. The enigmatic nature of the *L* mutation, however, only allows speculations as to the molecular basis of this interaction.

## Materials and methods

The following fly stocks were used: Oregon R (OreR) and *y*^*1*^ *w*^*67c23*^ (BL6599), *L*^*1*^ (BL318), *L*^*2*^ (BL319), *L*^*5*^ (BL321), *L*^*G*^ (BL322), *PKD*^*26*^ [40]; *ey*-Gal4 [42], UAS-PKD-WT, UAS-PKD-SE, UAS-PKD-kd [36]. Further information on fly strains is available at flybase.org. Flies were raised under non-crowded conditions on standard agar-corn-molasses food at 18 °C or 25 °C as indicated. Crosses and combinations were performed with standard genetic techniques; analyses were performed on one to 5 days old flies. Presence of the *PKD*^*26*^ allele in the recombinants was confirmed by PCR using the primer pair: P6 Cre-lox LP, 5′ CCG GAC AGT GGA CTC ACA TA 3′ and P8 white UP, 5′ AAA AGT GCA GCG GAA ATA GTT A 3′ [40]. Microphotographs of adult heads were taken with a Pixera ES120 digital camera (Optronics) coupled to a Leica M5 using the Pixera Viewfinder Version 2.0 software. Figures were assembled using *Corel Photo Paint, Corel Draw, Exel,* and *BoxPlotR* software. Statistical significance of probes was determined by ANOVA two-tailed test for multiple comparisons using Dunnet’s approach with *p*-values: *p* > 0.05 (not significant); *p* < 0.05; *p* < 0.01; *p* < 0.001.

## Data Availability

The datasets supporting the conclusions of this article are included within the article.
